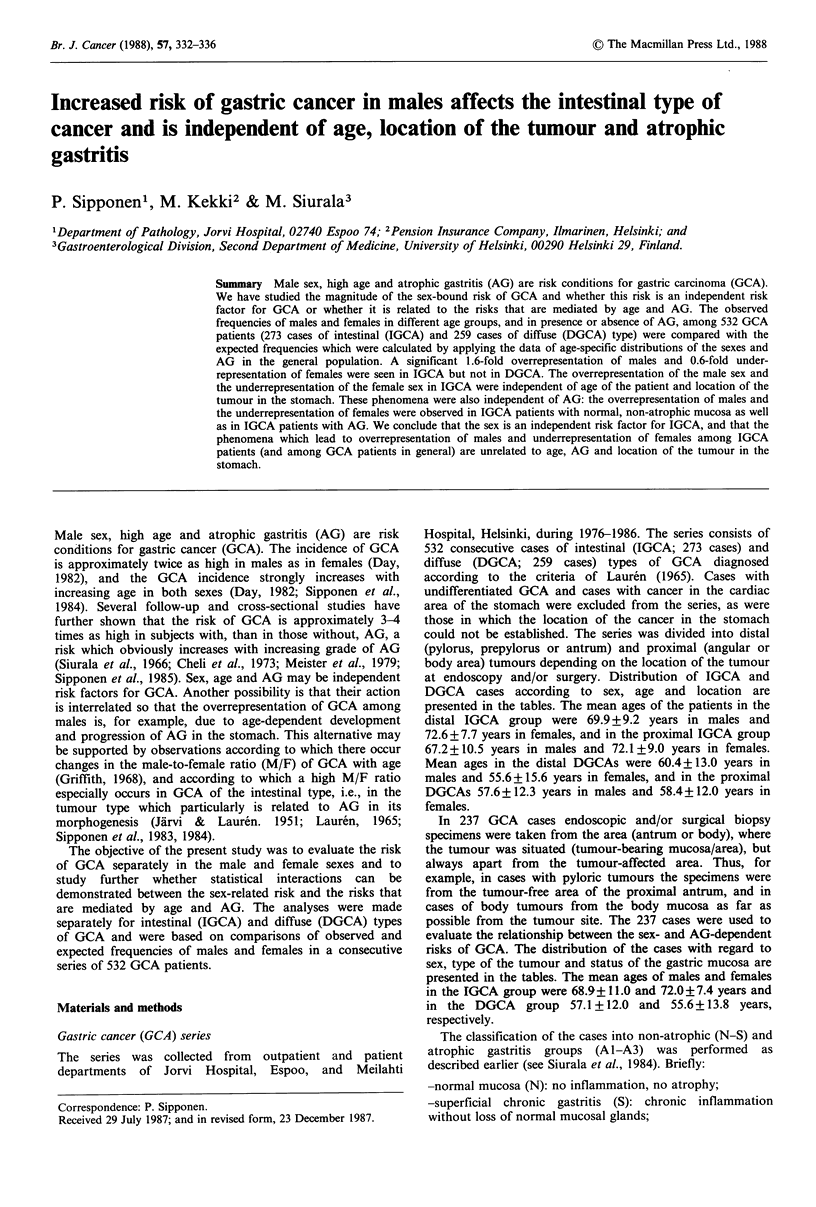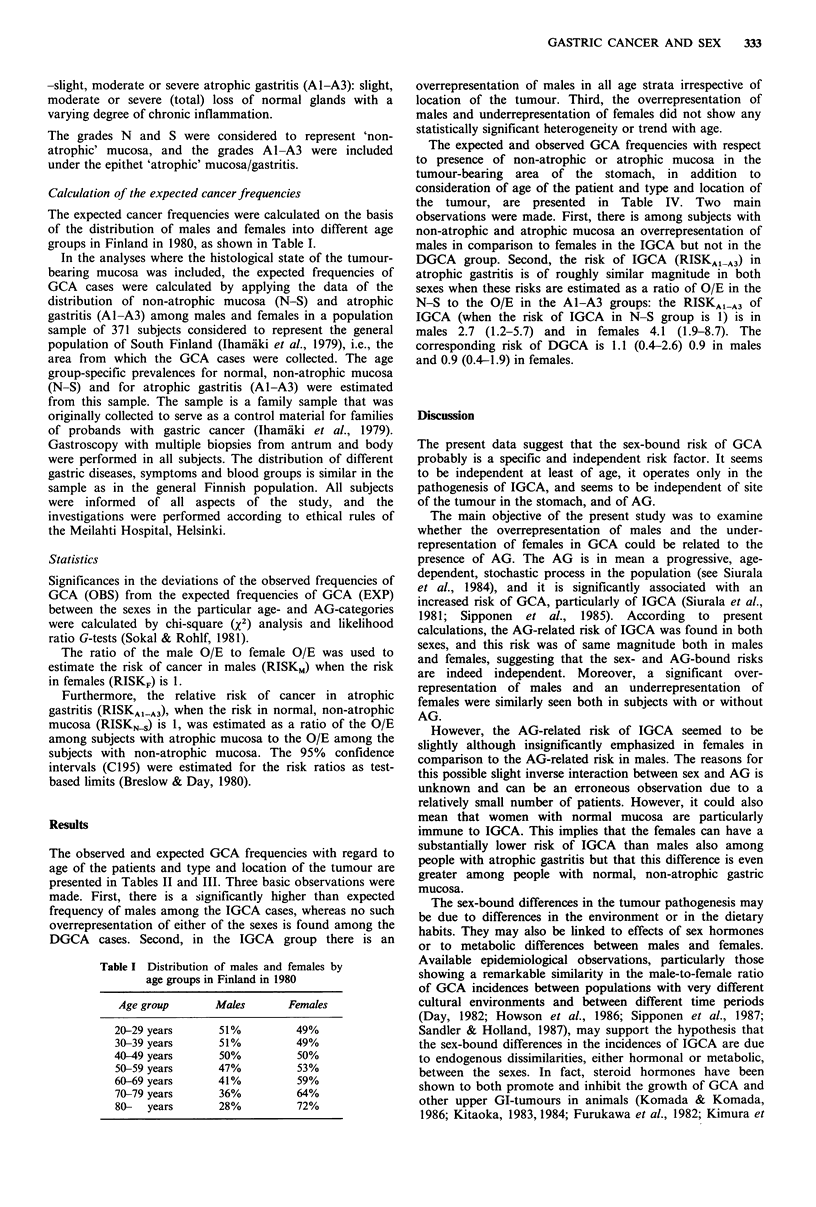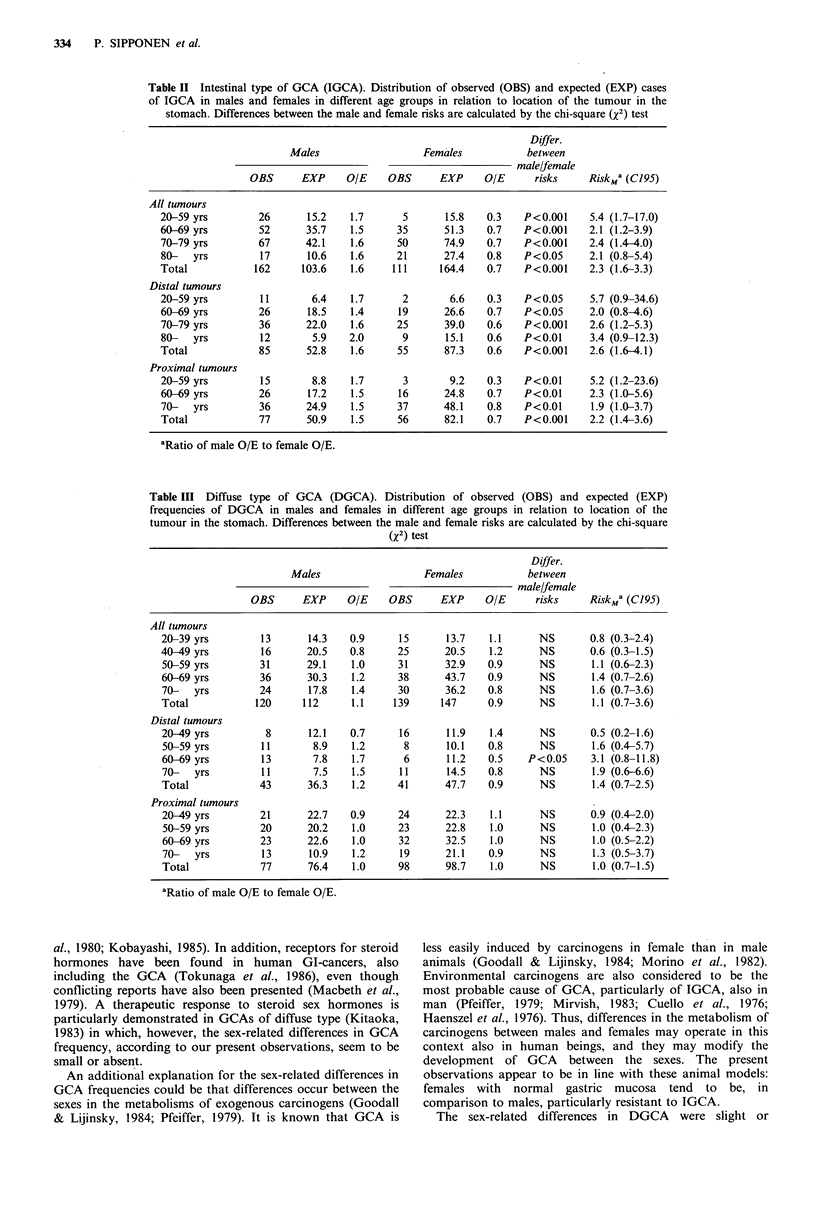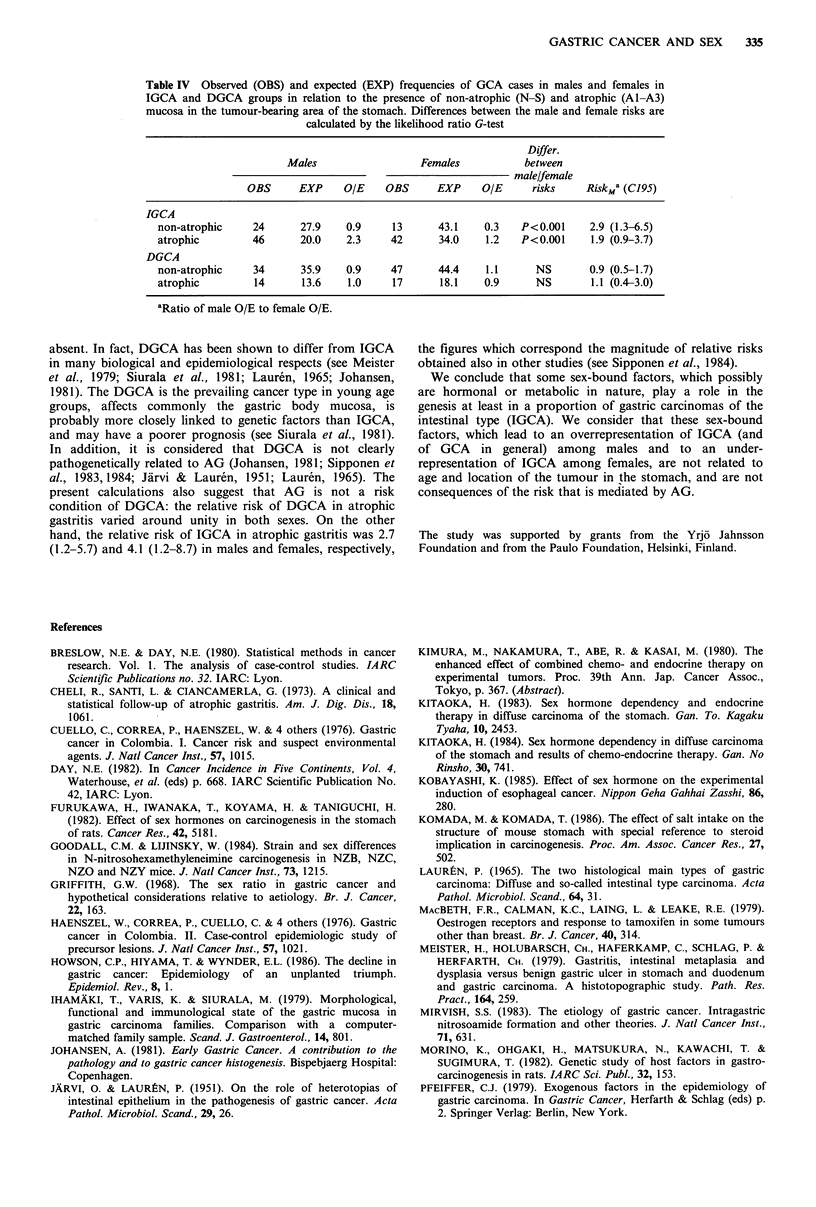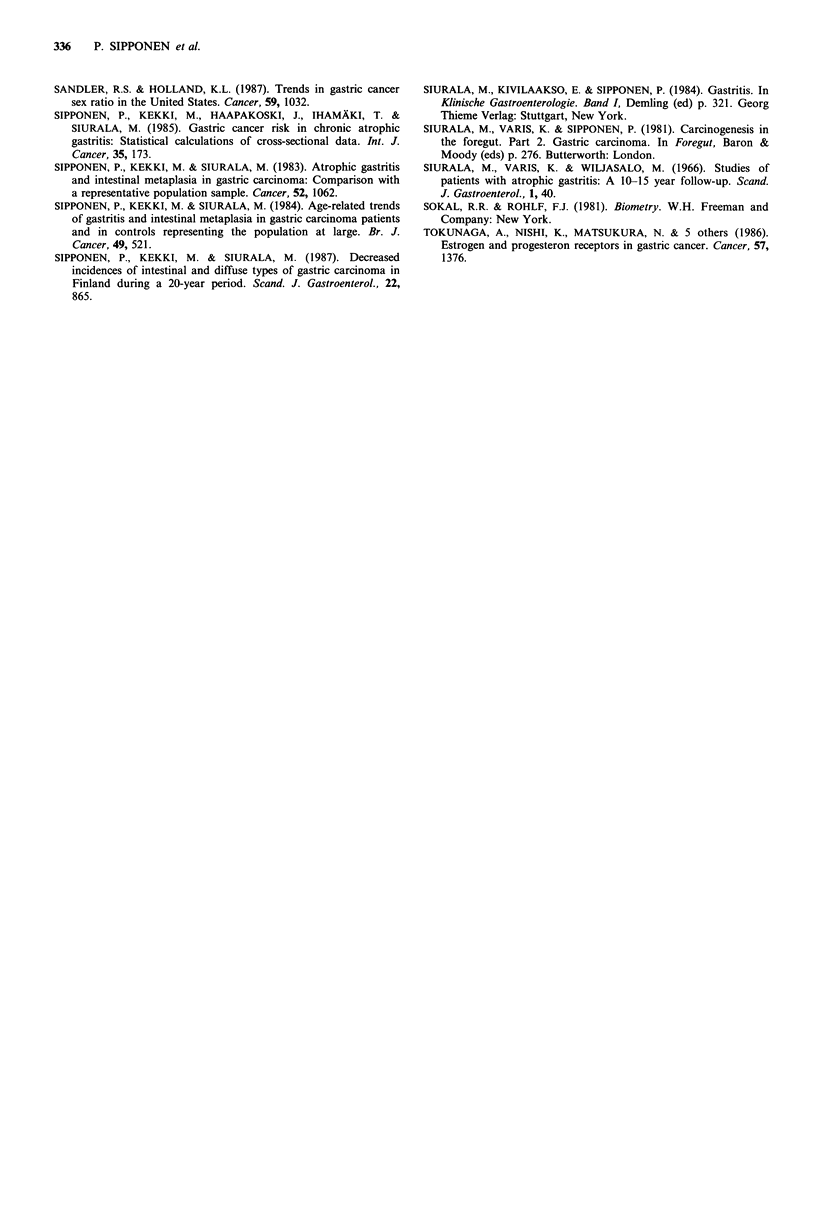# Increased risk of gastric cancer in males affects the intestinal type of cancer and is independent of age, location of the tumour and atrophic gastritis.

**DOI:** 10.1038/bjc.1988.75

**Published:** 1988-03

**Authors:** P. Sipponen, M. Kekki, M. Siurala

**Affiliations:** Department of Pathology, Jorvi Hospital, Espoo, Finland.

## Abstract

Male sex, high age and atrophic gastritis (AG) are risk conditions for gastric carcinoma (GCA). We have studied the magnitude of the sex-bound risk of GCA and whether this risk is an independent risk factor for GCA or whether it is related to the risks that are mediated by age and AG. The observed frequencies of males and females in different age groups, and in presence or absence of AG, among 532 GCA patients (273 cases of intestinal (IGCA) and 259 cases of diffuse (DGCA) type) were compared with the expected frequencies which were calculated by applying the data of age-specific distributions of the sexes and AG in the general population. A significant 1.6-fold overrepresentation of males and 0.6-fold underrepresentation of females were seen in IGCA but not in DGCA. The overrepresentation of the male sex and the underrepresentation of the female sex in IGCA were independent of age of the patient and location of the tumour in the stomach. These phenomena were also independent of AG: the overrepresentation of males and the underrepresentation of females were observed in IGCA patients with normal, non-atrophic mucosa as well as in IGCA patients with AG. We conclude that the sex is an independent risk factor for IGCA, and that the phenomena which lead to overrepresentation of males and underrepresentation of females among IGCA patients (and among GCA patients in general) are unrelated to age, AG and location of the tumour in the stomach.


*Br. J. Cancer* (1988), **57**, 332–336 © The Macmillan Press Ltd., 1988

### Increased risk of gastric cancer in males affects the intestinal type of cancer and is independent of age, location of the tumour and atrophic gastritis

P. Sipponen[1], M. Kekki[2] & M. Siurala[3]

[1]*Department of Pathology, Jorvi Hospital, 02740 Espoo 74;* [2]*Pension Insurance Company, Ilmarinen, Helsinki; and* [3]*Gastroenterological Division, Second Department of Medicine, University of Helsinki, 00290 Helsinki 29, Finland.*

**Summary** Male sex, high age and atrophic gastritis (AG) are risk conditions for gastric carcinoma (GCA). We have studied the magnitude of the sex-bound risk of GCA and whether this risk is an independent risk factor for GCA or whether it is related to the risks that are mediated by age and AG. The observed frequencies of males and females in different age groups, and in presence or absence of AG, among 532 GCA patients (273 cases of intestinal (IGCA) and 259 cases of diffuse (DGCA) type) were compared with the expected frequencies which were calculated by applying the data of age-specific distributions of the sexes and AG in the general population. A significant 1.6-fold overrepresentation of males and 0.6-fold underrepresentation of females were seen in IGCA but not in DGCA. The overrepresentation of the male sex and the underrepresentation of the female sex in IGCA were independent of age of the patient and location of the tumour in the stomach. These phenomena were also independent of AG: the overrepresentation of males and the underrepresentation of females were observed in IGCA patients with normal, non-atrophic mucosa as well as in IGCA patients with AG. We conclude that the sex is an independent risk factor for IGCA, and that the phenomena which lead to overrepresentation of males and underrepresentation of females among IGCA patients (and among GCA patients in general) are unrelated to age, AG and location of the tumour in the stomach.

Male sex, high age and atrophic gastritis (AG) are risk conditions for gastric cancer (GCA). The incidence of GCA is approximately twice as high in males as in females (Day, 1982), and the GCA incidence strongly increases with increasing age in both sexes (Day, 1982; Sipponen *et al.*, 1984). Several follow-up and cross-sectional studies have further shown that the risk of GCA is approximately 3–4 times as high in subjects with, than in those without, AG, a risk which obviously increases with increasing grade of AG (Siurala *et al.*, 1966; Cheli *et al.*, 1973; Meister *et al.*, 1979; Sipponen *et al.*, 1985). Sex, age and AG may be independent risk factors for GCA. Another possibility is that their action is interrelated so that the overrepresentation of GCA among males is, for example, due to age-dependent development and progression of AG in the stomach. This alternative may be supported by observations according to which there occur changes in the male-to-female ratio (M/F) of GCA with age (Griffith, 1968), and according to which a high M/F ratio especially occurs in GCA of the intestinal type, i.e., in the tumour type which particularly is related to AG in its morphogenesis (Järvi & Laurén. 1951; Laurén, 1965; Sipponen *et al.*, 1983, 1984).

The objective of the present study was to evaluate the risk of GCA separately in the male and female sexes and to study further whether statistical interactions can be demonstrated between the sex-related risk and the risks that are mediated by age and AG. The analyses were made separately for intestinal (IGCA) and diffuse (DGCA) types of GCA and were based on comparisons of observed and expected frequencies of males and females in a consecutive series of 532 GCA patients.

### Materials and methods

### *Gastric cancer (GCA) series*

The series was collected from outpatient and patient departments of Jorvi Hospital, Espoo, and Meilahti Hospital, Helsinki, during 1976–1986. The series consists of 532 consecutive cases of intestinal (IGCA; 273 cases) and diffuse (DGCA; 259 cases) types of GCA diagnosed according to the criteria of Laurén (1965). Cases with undifferentiated GCA and cases with cancer in the cardiac area of the stomach were excluded from the series, as were those in which the location of the cancer in the stomach could not be established. The series was divided into distal (pylorus, prepylorus or antrum) and proximal (angular or body area) tumours depending on the location of the tumour at endoscopy and/or surgery. Distribution of IGCA and DGCA cases according to sex, age and location are presented in the tables. The mean ages of the patients in the distal IGCA group were $69.9 \pm 9.2$ years in males and $72.6 \pm 7.7$ years in females, and in the proximal IGCA group $67.2 \pm 10.5$ years in males and $72.1 \pm 9.0$ years in females. Mean ages in the distal DGCAs were $60.4 \pm 13.0$ years in males and $55.6 \pm 15.6$ years in females, and in the proximal DGCAs $57.6 \pm 12.3$ years in males and $58.4 \pm 12.0$ years in females.

In 237 GCA cases endoscopic and/or surgical biopsy specimens were taken from the area (antrum or body), where the tumour was situated (tumour-bearing mucosa/area), but always apart from the tumour-affected area. Thus, for example, in cases with pyloric tumours the specimens were from the tumour-free area of the proximal antrum, and in cases of body tumours from the body mucosa as far as possible from the tumour site. The 237 cases were used to evaluate the relationship between the sex- and AG-dependent risks of GCA. The distribution of the cases with regard to sex, type of the tumour and status of the gastric mucosa are presented in the tables. The mean ages of males and females in the IGCA group were $68.9 \pm 11.0$ and $72.0 \pm 7.4$ years and in the DGCA group $57.1 \pm 12.0$ and $55.6 \pm 13.8$ years, respectively.

The classification of the cases into non-atrophic (N–S) and atrophic gastritis groups (A1–A3) was performed as described earlier (see Siurala *et al.*, 1984). Briefly:

–normal mucosa (N): no inflammation, no atrophy;

–superficial chronic gastritis (S): chronic inflammation without loss of normal mucosal glands;

Correspondence: P. Sipponen.
Received 29 July 1987; and in revised form, 23 December 1987.

–slight, moderate or severe atrophic gastritis (A1–A3): slight, moderate or severe (total) loss of normal glands with a varying degree of chronic inflammation.

The grades N and S were considered to represent 'non-atrophic' mucosa, and the grades A1–A3 were included under the epithet 'atrophic' mucosa/gastritis.

*Calculation of the expected cancer frequencies*

The expected cancer frequencies were calculated on the basis of the distribution of males and females into different age groups in Finland in 1980, as shown in Table I.

In the analyses where the histological state of the tumour-bearing mucosa was included, the expected frequencies of GCA cases were calculated by applying the data of the distribution of non-atrophic mucosa (N–S) and atrophic gastritis (A1–A3) among males and females in a population sample of 371 subjects considered to represent the general population of South Finland (Ihamäki *et al.*, 1979), i.e., the area from which the GCA cases were collected. The age group-specific prevalences for normal, non-atrophic mucosa (N–S) and for atrophic gastritis (A1–A3) were estimated from this sample. The sample is a family sample that was originally collected to serve as a control material for families of probands with gastric cancer (Ihamäki *et al.*, 1979). Gastroscopy with multiple biopsies from antrum and body were performed in all subjects. The distribution of different gastric diseases, symptoms and blood groups is similar in the sample as in the general Finnish population. All subjects were informed of all aspects of the study, and the investigations were performed according to ethical rules of the Meilahti Hospital, Helsinki.

*Statistics*

Significances in the deviations of the observed frequencies of GCA (OBS) from the expected frequencies of GCA (EXP) between the sexes in the particular age- and AG-categories were calculated by chi-square ($\chi^2$) analysis and likelihood ratio *G*-tests (Sokal & Rohlf, 1981).

The ratio of the male O/E to female O/E was used to estimate the risk of cancer in males ($RISK_M$) when the risk in females ($RISK_F$) is 1.

Furthermore, the relative risk of cancer in atrophic gastritis ($RISK_{A1-A3}$), when the risk in normal, non-atrophic mucosa ($RISK_{N-S}$) is 1, was estimated as a ratio of the O/E among subjects with atrophic mucosa to the O/E among the subjects with non-atrophic mucosa. The 95% confidence intervals (C195) were estimated for the risk ratios as test-based limits (Breslow & Day, 1980).

### Results

The observed and expected GCA frequencies with regard to age of the patients and type and location of the tumour are presented in Tables II and III. Three basic observations were made. First, there is a significantly higher than expected frequency of males among the IGCA cases, whereas no such overrepresentation of either of the sexes is found among the DGCA cases. Second, in the IGCA group there is an overrepresentation of males in all age strata irrespective of location of the tumour. Third, the overrepresentation of males and underrepresentation of females did not show any statistically significant heterogeneity or trend with age.

**Table I** Distribution of males and females by age groups in Finland in 1980

| *Age group* | *Males* | *Females* |
|---|---|---|
| 20–29 years | 51% | 49% |
| 30–39 years | 51% | 49% |
| 40–49 years | 50% | 50% |
| 50–59 years | 47% | 53% |
| 60–69 years | 41% | 59% |
| 70–79 years | 36% | 64% |
| 80– years | 28% | 72% |

The expected and observed GCA frequencies with respect to presence of non-atrophic or atrophic mucosa in the tumour-bearing area of the stomach, in addition to consideration of age of the patient and type and location of the tumour, are presented in Table IV. Two main observations were made. First, there is among subjects with non-atrophic and atrophic mucosa an overrepresentation of males in comparison to females in the IGCA but not in the DGCA group. Second, the risk of IGCA ($RISK_{A1-A3}$) in atrophic gastritis is of roughly similar magnitude in both sexes when these risks are estimated as a ratio of O/E in the N–S to the O/E in the A1–A3 groups: the $RISK_{A1-A3}$ of IGCA (when the risk of IGCA in N–S group is 1) is in males 2.7 (1.2–5.7) and in females 4.1 (1.9–8.7). The corresponding risk of DGCA is 1.1 (0.4–2.6) 0.9 in males and 0.9 (0.4–1.9) in females.

### Discussion

The present data suggest that the sex-bound risk of GCA probably is a specific and independent risk factor. It seems to be independent at least of age, it operates only in the pathogenesis of IGCA, and seems to be independent of site of the tumour in the stomach, and of AG.

The main objective of the present study was to examine whether the overrepresentation of males and the under-representation of females in GCA could be related to the presence of AG. The AG is in mean a progressive, age-dependent, stochastic process in the population (see Siurala *et al.*, 1984), and it is significantly associated with an increased risk of GCA, particularly of IGCA (Siurala *et al.*, 1981; Sipponen *et al.*, 1985). According to present calculations, the AG-related risk of IGCA was found in both sexes, and this risk was of same magnitude both in males and females, suggesting that the sex- and AG-bound risks are indeed independent. Moreover, a significant over-representation of males and an underrepresentation of females were similarly seen both in subjects with or without AG.

However, the AG-related risk of IGCA seemed to be slightly although insignificantly emphasized in females in comparison to the AG-related risk in males. The reasons for this possible slight inverse interaction between sex and AG is unknown and can be an erroneous observation due to a relatively small number of patients. However, it could also mean that women with normal mucosa are particularly immune to IGCA. This implies that the females can have a substantially lower risk of IGCA than males also among people with atrophic gastritis but that this difference is even greater among people with normal, non-atrophic gastric mucosa.

The sex-bound differences in the tumour pathogenesis may be due to differences in the environment or in the dietary habits. They may also be linked to effects of sex hormones or to metabolic differences between males and females. Available epidemiological observations, particularly those showing a remarkable similarity in the male-to-female ratio of GCA incidences between populations with very different cultural environments and between different time periods (Day, 1982; Howson *et al.*, 1986; Sipponen *et al.*, 1987; Sandler & Holland, 1987), may support the hypothesis that the sex-bound differences in the incidences of IGCA are due to endogenous dissimilarities, either hormonal or metabolic, between the sexes. In fact, steroid hormones have been shown to both promote and inhibit the growth of GCA and other upper GI-tumours in animals (Komada & Komada, 1986; Kitaoka, 1983, 1984; Furukawa *et al.*, 1982; Kimura *et*

**Table II** Intestinal type of GCA (IGCA). Distribution of observed (OBS) and expected (EXP) cases of IGCA in males and females in different age groups in relation to location of the tumour in the stomach. Differences between the male and female risks are calculated by the chi-square ($\chi^2$) test

| | *Males* | | | *Females* | | | *Differ. between male/female risks* | |
|---|---|---|---|---|---|---|---|---|
| | *OBS* | *EXP* | *O/E* | *OBS* | *EXP* | *O/E* | | *Risk*$_M$[a] *(CI95)* |
| *All tumours* | | | | | | | | |
| 20–59 yrs | 26 | 15.2 | 1.7 | 5 | 15.8 | 0.3 | $P<0.001$ | 5.4 (1.7–17.0) |
| 60–69 yrs | 52 | 35.7 | 1.5 | 35 | 51.3 | 0.7 | $P<0.001$ | 2.1 (1.2–3.9) |
| 70–79 yrs | 67 | 42.1 | 1.6 | 50 | 74.9 | 0.7 | $P<0.001$ | 2.4 (1.4–4.0) |
| 80– yrs | 17 | 10.6 | 1.6 | 21 | 27.4 | 0.8 | $P<0.05$ | 2.1 (0.8–5.4) |
| Total | 162 | 103.6 | 1.6 | 111 | 164.4 | 0.7 | $P<0.001$ | 2.3 (1.6–3.3) |
| *Distal tumours* | | | | | | | | |
| 20–59 yrs | 11 | 6.4 | 1.7 | 2 | 6.6 | 0.3 | $P<0.05$ | 5.7 (0.9–34.6) |
| 60–69 yrs | 26 | 18.5 | 1.4 | 19 | 26.6 | 0.7 | $P<0.05$ | 2.0 (0.8–4.6) |
| 70–79 yrs | 36 | 22.0 | 1.6 | 25 | 39.0 | 0.6 | $P<0.001$ | 2.6 (1.2–5.3) |
| 80– yrs | 12 | 5.9 | 2.0 | 9 | 15.1 | 0.6 | $P<0.01$ | 3.4 (0.9–12.3) |
| Total | 85 | 52.8 | 1.6 | 55 | 87.3 | 0.6 | $P<0.001$ | 2.6 (1.6–4.1) |
| *Proximal tumours* | | | | | | | | |
| 20–59 yrs | 15 | 8.8 | 1.7 | 3 | 9.2 | 0.3 | $P<0.01$ | 5.2 (1.2–23.6) |
| 60–69 yrs | 26 | 17.2 | 1.5 | 16 | 24.8 | 0.7 | $P<0.01$ | 2.3 (1.0–5.6) |
| 70– yrs | 36 | 24.9 | 1.5 | 37 | 48.1 | 0.8 | $P<0.01$ | 1.9 (1.0–3.7) |
| Total | 77 | 50.9 | 1.5 | 56 | 82.1 | 0.7 | $P<0.001$ | 2.2 (1.4–3.6) |

[a]Ratio of male O/E to female O/E.

**Table III** Diffuse type of GCA (DGCA). Distribution of observed (OBS) and expected (EXP) frequencies of DGCA in males and females in different age groups in relation to location of the tumour in the stomach. Differences between the male and female risks are calculated by the chi-square ($\chi^2$) test

| | *Males* | | | *Females* | | | *Differ. between male/female risks* | |
|---|---|---|---|---|---|---|---|---|
| | *OBS* | *EXP* | *O/E* | *OBS* | *EXP* | *O/E* | | *Risk*$_M$[a] *(CI95)* |
| *All tumours* | | | | | | | | |
| 20–39 yrs | 13 | 14.3 | 0.9 | 15 | 13.7 | 1.1 | NS | 0.8 (0.3–2.4) |
| 40–49 yrs | 16 | 20.5 | 0.8 | 25 | 20.5 | 1.2 | NS | 0.6 (0.3–1.5) |
| 50–59 yrs | 31 | 29.1 | 1.0 | 31 | 32.9 | 0.9 | NS | 1.1 (0.6–2.3) |
| 60–69 yrs | 36 | 30.3 | 1.2 | 38 | 43.7 | 0.9 | NS | 1.4 (0.7–2.6) |
| 70– yrs | 24 | 17.8 | 1.4 | 30 | 36.2 | 0.8 | NS | 1.6 (0.7–3.6) |
| Total | 120 | 112 | 1.1 | 139 | 147 | 0.9 | NS | 1.1 (0.7–3.6) |
| *Distal tumours* | | | | | | | | |
| 20–49 yrs | 8 | 12.1 | 0.7 | 16 | 11.9 | 1.4 | NS | 0.5 (0.2–1.6) |
| 50–59 yrs | 11 | 8.9 | 1.2 | 8 | 10.1 | 0.8 | NS | 1.6 (0.4–5.7) |
| 60–69 yrs | 13 | 7.8 | 1.7 | 6 | 11.2 | 0.5 | $P<0.05$ | 3.1 (0.8–11.8) |
| 70– yrs | 11 | 7.5 | 1.5 | 11 | 14.5 | 0.8 | NS | 1.9 (0.6–6.6) |
| Total | 43 | 36.3 | 1.2 | 41 | 47.7 | 0.9 | NS | 1.4 (0.7–2.5) |
| *Proximal tumours* | | | | | | | | |
| 20–49 yrs | 21 | 22.7 | 0.9 | 24 | 22.3 | 1.1 | NS | 0.9 (0.4–2.0) |
| 50–59 yrs | 20 | 20.2 | 1.0 | 23 | 22.8 | 1.0 | NS | 1.0 (0.4–2.3) |
| 60–69 yrs | 23 | 22.6 | 1.0 | 32 | 32.5 | 1.0 | NS | 1.0 (0.5–2.2) |
| 70– yrs | 13 | 10.9 | 1.2 | 19 | 21.1 | 0.9 | NS | 1.3 (0.5–3.7) |
| Total | 77 | 76.4 | 1.0 | 98 | 98.7 | 1.0 | NS | 1.0 (0.7–1.5) |

[a]Ratio of male O/E to female O/E.

*al.*, 1980; Kobayashi, 1985). In addition, receptors for steroid hormones have been found in human GI-cancers, also including the GCA (Tokunaga *et al.*, 1986), even though conflicting reports have also been presented (Macbeth *et al.*, 1979). A therapeutic response to steroid sex hormones is particularly demonstrated in GCAs of diffuse type (Kitaoka, 1983) in which, however, the sex-related differences in GCA frequency, according to our present observations, seem to be small or absent.

An additional explanation for the sex-related differences in GCA frequencies could be that differences occur between the sexes in the metabolisms of exogenous carcinogens (Goodall & Lijinsky, 1984; Pfeiffer, 1979). It is known that GCA is less easily induced by carcinogens in female than in male animals (Goodall & Lijinsky, 1984; Morino *et al.*, 1982). Environmental carcinogens are also considered to be the most probable cause of GCA, particularly of IGCA, also in man (Pfeiffer, 1979; Mirvish, 1983; Cuello *et al.*, 1976; Haenszel *et al.*, 1976). Thus, differences in the metabolism of carcinogens between males and females may operate in this context also in human beings, and they may modify the development of GCA between the sexes. The present observations appear to be in line with these animal models: females with normal gastric mucosa tend to be, in comparison to males, particularly resistant to IGCA.

The sex-related differences in DGCA were slight or

**Table IV** Observed (OBS) and expected (EXP) frequencies of GCA cases in males and females in IGCA and DGCA groups in relation to the presence of non-atrophic (N–S) and atrophic (A1–A3) mucosa in the tumour-bearing area of the stomach. Differences between the male and female risks are calculated by the likelihood ratio *G*-test

| | *Males* | | | *Females* | | | *Differ. between male/female risks* | |
|---|---|---|---|---|---|---|---|---|
| | *OBS* | *EXP* | *O/E* | *OBS* | *EXP* | *O/E* | | *Risk*$_M$[a] *(CI95)* |
| *IGCA* | | | | | | | | |
| non-atrophic | 24 | 27.9 | 0.9 | 13 | 43.1 | 0.3 | $P<0.001$ | 2.9 (1.3–6.5) |
| atrophic | 46 | 20.0 | 2.3 | 42 | 34.0 | 1.2 | $P<0.001$ | 1.9 (0.9–3.7) |
| *DGCA* | | | | | | | | |
| non-atrophic | 34 | 35.9 | 0.9 | 47 | 44.4 | 1.1 | NS | 0.9 (0.5–1.7) |
| atrophic | 14 | 13.6 | 1.0 | 17 | 18.1 | 0.9 | NS | 1.1 (0.4–3.0) |

[a]Ratio of male O/E to female O/E.

absent. In fact, DGCA has been shown to differ from IGCA in many biological and epidemiological respects (see Meister *et al.*, 1979; Siurala *et al.*, 1981; Laurén, 1965; Johansen, 1981). The DGCA is the prevailing cancer type in young age groups, affects commonly the gastric body mucosa, is probably more closely linked to genetic factors than IGCA, and may have a poorer prognosis (see Siurala *et al.*, 1981). In addition, it is considered that DGCA is not clearly pathogenetically related to AG (Johansen, 1981; Sipponen *et al.*, 1983, 1984; Järvi & Laurén, 1951; Laurén, 1965). The present calculations also suggest that AG is not a risk condition of DGCA: the relative risk of DGCA in atrophic gastritis varied around unity in both sexes. On the other hand, the relative risk of IGCA in atrophic gastritis was 2.7 (1.2–5.7) and 4.1 (1.2–8.7) in males and females, respectively, the figures which correspond the magnitude of relative risks obtained also in other studies (see Sipponen *et al.*, 1984).

We conclude that some sex-bound factors, which possibly are hormonal or metabolic in nature, play a role in the genesis at least in a proportion of gastric carcinomas of the intestinal type (IGCA). We consider that these sex-bound factors, which lead to an overrepresentation of IGCA (and of GCA in general) among males and to an under-representation of IGCA among females, are not related to age and location of the tumour in the stomach, and are not consequences of the risk that is mediated by AG.

The study was supported by grants from the Yrjö Jahnsson Foundation and from the Paulo Foundation, Helsinki, Finland.

### References

BRESLOW, N.E. & DAY, N.E. (1980). Statistical methods in cancer research. Vol. 1. The analysis of case-control studies. *IARC Scientific Publications no. 32.* IARC: Lyon.

CHELI, R., SANTI, L. & CIANCAMERLA, G. (1973). A clinical and statistical follow-up of atrophic gastritis. *Am. J. Dig. Dis.*, **18**, 1061.

CUELLO, C., CORREA, P., HAENSZEL, W. & 4 others (1976). Gastric cancer in Colombia. I. Cancer risk and suspect environmental agents. *J. Natl Cancer Inst.*, **57**, 1015.

DAY, N.E. (1982). In *Cancer Incidence in Five Continents, Vol. 4,* Waterhouse, *et al.* (eds) p. 668. IARC Scientific Publication No. 42, IARC: Lyon.

FURUKAWA, H., IWANAKA, T., KOYAMA, H. & TANIGUCHI, H. (1982). Effect of sex hormones on carcinogenesis in the stomach of rats. *Cancer Res.*, **42**, 5181.

GOODALL, C.M. & LIJINSKY, W. (1984). Strain and sex differences in N-nitrosohexamethyleneimine carcinogenesis in NZB, NZC, NZO and NZY mice. *J. Natl Cancer Inst.*, **73**, 1215.

GRIFFITH, G.W. (1968). The sex ratio in gastric cancer and hypothetical considerations relative to aetiology. *Br. J. Cancer*, **22**, 163.

HAENSZEL, W., CORREA, P., CUELLO, C. & 4 others (1976). Gastric cancer in Colombia. II. Case-control epidemiologic study of precursor lesions. *J. Natl Cancer Inst.*, **57**, 1021.

HOWSON, C.P., HIYAMA, T. & WYNDER, E.L. (1986). The decline in gastric cancer: Epidemiology of an unplanted triumph. *Epidemiol. Rev.*, **8**, 1.

IHAMÄKI, T., VARIS, K. & SIURALA, M. (1979). Morphological, functional and immunological state of the gastric mucosa in gastric carcinoma families. Comparison with a computer-matched family sample. *Scand. J. Gastroenterol.*, **14**, 801.

JOHANSEN, A. (1981). *Early Gastric Cancer. A contribution to the pathology and to gastric cancer histogenesis.* Bispebjaerg Hospital: Copenhagen.

JÄRVI, O. & LAURÉN, P. (1951). On the role of heterotopias of intestinal epithelium in the pathogenesis of gastric cancer. *Acta Pathol. Microbiol. Scand.*, **29**, 26.

KIMURA, M., NAKAMURA, T., ABE, R. & KASAI, M. (1980). The enhanced effect of combined chemo- and endocrine therapy on experimental tumors. Proc. 39th Ann. Jap. Cancer Assoc., Tokyo, p. 367. (*Abstract*).

KITAOKA, H. (1983). Sex hormone dependency and endocrine therapy in diffuse carcinoma of the stomach. *Gan. To. Kagaku Tyaha,* **10**, 2453.

KITAOKA, H. (1984). Sex hormone dependency in diffuse carcinoma of the stomach and results of chemo-endocrine therapy. *Gan. No Rinsho*, **30**, 741.

KOBAYASHI, K. (1985). Effect of sex hormone on the experimental induction of esophageal cancer. *Nippon Geha Gahhai Zasshi*, **86**, 280.

KOMADA, M. & KOMADA, T. (1986). The effect of salt intake on the structure of mouse stomach with special reference to steroid implication in carcinogenesis. *Proc. Am. Assoc. Cancer Res.*, **27**, 502.

LAURÉN, P. (1965). The two histological main types of gastric carcinoma: Diffuse and so-called intestinal type carcinoma. *Acta Pathol. Microbiol. Scand.*, **64**, 31.

MACBETH, F.R., CALMAN, K.C., LAING, L. & LEAKE, R.E. (1979). Oestrogen receptors and response to tamoxifen in some tumours other than breast. *Br. J. Cancer*, **40**, 314.

MEISTER, H., HOLUBARSCH, CH., HAFERKAMP, C., SCHLAG, P. & HERFARTH, CH. (1979). Gastritis, intestinal metaplasia and dysplasia versus benign gastric ulcer in stomach and duodenum and gastric carcinoma. A histotopographic study. *Path. Res. Pract.*, **164**, 259.

MIRVISH, S.S. (1983). The etiology of gastric cancer. Intragastric nitrosoamide formation and other theories. *J. Natl Cancer Inst.*, **71**, 631.

MORINO, K., OHGAKI, H., MATSUKURA, N., KAWACHI, T. & SUGIMURA, T. (1982). Genetic study of host factors in gastro-carcinogenesis in rats. *IARC Sci. Publ.*, **32**, 153.

PFEIFFER, C.J. (1979). Exogenous factors in the epidemiology of gastric carcinoma. In *Gastric Cancer*, Herfarth & Schlag (eds) p. 2. Springer Verlag: Berlin, New York.

SANDLER, R.S. & HOLLAND, K.L. (1987). Trends in gastric cancer sex ratio in the United States. *Cancer*, **59**, 1032.

SIPPONEN, P., KEKKI, M., HAAPAKOSKI, J., IHAMÄKI, T. & SIURALA, M. (1985). Gastric cancer risk in chronic atrophic gastritis: Statistical calculations of cross-sectional data. *Int. J. Cancer*, **35**, 173.

SIPPONEN, P., KEKKI, M. & SIURALA, M. (1983). Atrophic gastritis and intestinal metaplasia in gastric carcinoma: Comparison with a representative population sample. *Cancer*, **52**, 1062.

SIPPONEN, P., KEKKI, M. & SIURALA, M. (1984). Age-related trends of gastritis and intestinal metaplasia in gastric carcinoma patients and in controls representing the population at large. *Br. J. Cancer*, **49**, 521.

SIPPONEN, P., KEKKI, M. & SIURALA, M. (1987). Decreased incidences of intestinal and diffuse types of gastric carcinoma in Finland during a 20-year period. *Scand. J. Gastroenterol.*, **22**, 865.

SIURALA, M., KIVILAAKSO, E. & SIPPONEN, P. (1984). Gastritis. In *Klinische Gastroenterologie. Band I*, Demling (ed) p. 321. Georg Thieme Verlag: Stuttgart, New York.

SIURALA, M., VARIS, K. & SIPPONEN, P. (1981). Carcinogenesis in the foregut. Part 2. Gastric carcinoma. In *Foregut*, Baron & Moody (eds) p. 276. Butterworth: London.

SIURALA, M., VARIS, K. & WILJASALO, M. (1966). Studies of patients with atrophic gastritis: A 10–15 year follow-up. *Scand. J. Gastroenterol.*, **1**, 40.

SOKAL, R.R. & ROHLF, F.J. (1981). *Biometry*. W.H. Freeman and Company: New York.

TOKUNAGA, A., NISHI, K., MATSUKURA, N. & 5 others (1986). Estrogen and progesteron receptors in gastric cancer. *Cancer*, **57**, 1376.